# A comparative study between Wuweizi seed and its post-ethanol extraction residue in normal and hypercholesterolemic mice

**DOI:** 10.1186/s12944-015-0097-z

**Published:** 2015-08-25

**Authors:** Zhu-Sheng Chu, Zhi-Ling Yu, Si-Yuan Pan, Zhan-Hong Jia, Xiao-Yan Wang, Yi Zhang, Pei-Li Zhu, Xiu-Juan Wang, Kam-Ming Ko

**Affiliations:** School of Chinese Materia Medica, Beijing University of Chinese Medicine, Beijing, 100102 China; School of Chinese Medicine, Hong Kong Baptist University, Hong Kong, China; School of Traditional Medicine, Capital Medical University, Beijing, 100069 China; Division of Life Science, Hong Kong University of Science & Technology, Hong Kong, China

**Keywords:** Schisandrae Chinensis Fructus seed, Fenofibrate, Hypercholesterolemic mice, Lipids, Glucose, Fecal cholesterol, Epididymal fat, Hepatomegaly

## Abstract

**Background:**

At the present, a shift from drug therapy, especially herbal therapy, to dietary supplementation is a trend in the management of dyslipidemia and related diseases. Therefore, the optimal utilization of herbal resource is important for a sustainable development of herbal medicine. Here, we compared the effects of dietary supplementation with Chinese medicine Schisandrae Chinensis Fructus seed (FSC-S) and the post-ethanol extraction residue of FSC-S (FSC-SpEt) on normal diet-fed (normal) and experimental hypercholesterolemic (HCL) mice.

**Methods:**

Male ICR mice (*n* = 10 in each group), weighing 17–21 g, were fed with normal diet (ND) or high cholesterol/bile salt (1/0.3 %, w/w) diet (HCBD) with or without supplemented with FSC-S, FSC-SpEt), or lipid-lowering agent fenofibrate (FF). Ten days later, serum/hepatic lipid and glucose (GLU) levels, body weight, organ/epididymal fat masses, and food/water intake were measured. Lipid level measurements included those of total cholesterol (TC), triglyceride (TG), high density lipoprotein (HDL), low density lipoprotein (LDL), HDL/LDL ratio, LDL/HDL ratio, and non-HDL (N-HDL).

**Results:**

Supplementation with FSC-S and FSC-SpEt increased serum TC (by 64 and 25 %, respectively) and LDL (by 60 and 27 %, respectively) in normal mice. FSC-S supplementation elevated serum TC, TG, HDL, LDL, and LDL/HDL ratio (up to 64, 118, 77, 197, and 51 %, respectively) in HCL mice. FSC-SpEt supplementation reduced serum TG (by 15 %) and LDL/HDL ratio (by 18 %), as well as increased serum HDL (by 22 %) and HDL/LDL ratio (by 21 %) in HCBD-fed mice. FSC-S decreased hepatic TC (by 19 %) contents and increased hepatic TG contents by 14 % in normal mice. FSC-S reduced hepatic GLU level in both normal and HCL mice by 24 and 22 %, respectively. Hepatic TC and TG contents were lowered in FSC-SpEt-supplemented normal mice by 16 and 20 %, respectively. The body/fatty masse and food intake were lowered, but the feed efficiency index (FEI), weight gain per unit of food ingested, was increased in FSC-S-supplemented normal and HCL mice. FF supplements reduced serum/hepatic lipids, hepatic GLU contents, and epididymal fat mass, but it induced hepatomegaly and high serum alanine aminotransferase (ALT) activity in normal and/or HCL mice.

**Conclusion:**

The ensemble of results indicated that while FSC-SpEt supplementation is beneficial for the treatment of hyperlipidemia/fatty liver, FSC-S is potentially useful for the management of overweight/obesity.

## Background

With dietary and lifestyle changes such as increases in high caloric food intake and sedentary lifestyle, there has been an appreciable rise in the prevalence of cardiovascular diseases (CVD) and obesity/overweight in developing countries [1, 2]. Among many pathogenetic factors, hyperlipidemia, especially hypertriglyceridemia, is considered as a significant risk factor for CVD, particularly the coronary heart disease [3, 4]. Therefore, reducing blood lipids and body mass is an important strategy for preventing the occurrence and progression of CVD. At present, drugs used for the treatment of hyperlipidemia include HMG CoA reductase inhibitors, fibrates, cholesterol absorption inhibitors, nicotinic acid group and bile acid sequestrants [5]. In the market, there are more than 10 prescription anti-obesity drugs such as Reductil, Xenical, Didrex, Tenuate, Belviq, Bontril, Adipex, Orlistat, and Qsymia, etc [6–8]. While most currently used lipid-lowering and weight-loss chemicals are either expensive or having potential side effects, recent research has been focused on exploring alternative natural medicines for the prevention and/or treatment of hyperlipidemia and/or obesity [9, 10]. However, a number of medicinal plants are facing the threat of extinction due to overexploitation [11, 12]. The situation can partly be attributed to the waste of natural herbal resources in pharmaceutical industry.

Schisandrae Chinensis Fructus (FSC, Wuweizi in Chinese), the fruit of *Schisandra chinensis* (Turcz.) Baill, is a commonly prescribed herb in traditional Chinese medicine, particularly in a number of tonic formulae. FSC has previously been reported to have a wide spectrum of biological effects, including protecting against chemically and virally induced hepatic injury [13, 14], improving insulin sensitivity [15], protecting against oxidative damage [16], producing sedative–hypnotic activity and anti-inflammatory effects [17, 18]. Our previous works have demonstrated that FSC extract and FSC-related compounds significantly altered lipid metabolism in mice [19–23]. In the present study, we endeavored to evaluate the effects of dietary supplementation with FSC seed (FSC-S) and the post-ethanol extraction residue of FSC-S (FSC-SpEt) on serum and hepatic lipid/glucose (GLU) contents in both normal diet-fed (ND) and high cholesterol/bile salt diet-fed (HCBD) mice, an animal model of hypercholesterolemia (HCL). Fenofibrate (FF), the most commonly prescribed lipid-lowering agent in Western medicine [24], was used as a positive control for comparison. The lipid profile measurements in the serum and liver samples included total cholesterol (TC), triglyceride (TG), and non-HDL (N-HDL), as well as high density lipoprotein (HDL), low density lipoprotein (LDL), HDL/LDL ratio, and LDL/HDL ratio.

## Results

### Effects of FSC-S and FSC-SpEt supplementation on serum lipid profiles and GLU

As shown in Table 1, feeding mice with FSC-S supplemented ND produced noticeable increases in serum TC (64 %), TG (155 %), HDL (68 %), and LDL (60 %) levels, but a decrease in serum GLU level by 32 %, when compared with the control group (i.e., un-supplemented ND). FSC-SpEt supplementation increased serum TC (by 25 %), LDL (27 %) and N-HDL (60 %) levels, as well as LDL/HDL ratio (15 %). However, it decreased the HDL/LDL ratio by 15 %. Daily supplementation with FF decreased serum TG level by 44 % in normal mice.

**Table 1:** Effects of FSC-S and FSC-SpEt supplementation on serum lipid profiles and GLU in normal and experimental hypercholesterolemic (HCL) mice

Groups	Dose	TC	TG	HDL	LDL	HDL/LDL	LDL/HDL	N-HDL	GLU
	(%, w/w)	(mmol/L)	(mmol/L)	(mmol/L)	(mmol/L)			(mmol/L)	(mmol/L)
*Normal mice*									
ND	-	3.58 ± 0.17	1.42 ± 0.21	2.41 ± 0.05	0.30 ± 0.01	8.06 ± 0.32	0.13 ± 0.01	1.17 ± 0.15	10.29 ± 0.50
ND/FSC-S	9	5.87 ± 0.34^**^	3.62 ± 0.31^**^	4.06 ± 0.11^**^	0.48 ± 0.02^**^	8.74 ± 0.50	0.12 ± 0.01	1.80 ± 0.37	7.03 ± 0.48^**^
ND/FSC-SpEt	9	4.46 ± 0.16^**^	1.22 ± 0.09	2.58 ± 0.09	0.38 ± 0.01^**^	6.86 ± 0.22^**^	0.15 ± 0.01^**^	1.87 ± 0.14^**^	11.47 ± 0.29
ND/FF	0.05	3.66 ± 0.30	0.79 ± 0.04^*^	2.36 ± 0.07	0.33 ± 0.02	7.40 ± 0.45	0.14 ± 0.01	1.29 ± 0.28	10.96 ± 0.41
*HCL mice*									
ND	-	4.31 ± 0.15	1.29 ± 0.04	2.96 ± 0.15	0.36 ± 0.03	8.67 ± 0.54	0.12 ± 0.01	1.35 ± 0.10	8.14 ± 0.36
HCBD	-	5.74 ± 0.19^**^	0.99 ± 0.04^**^	3.00 ± 0.08	1.50 ± 0.07^**^	2.05 ± 0.13^**^	0.50 ± 0.02^**^	2.73 ± 0.15^**^	8.00 ± 0.19
HCBD/FSC-S	3	6.98 ± 0.32^##^	1.23 ± 0.12	3.72 ± 0.14^##^	2.22 ± 0.08^##^	1.68 ± 0.05^#^	0.60 ± 0.02^##^	3.26 ± 0.21	7.78 ± 0.28
	9	9.41 ± 0.55^##^	2.16 ± 0.29^##^	5.30 ± 0.24^##^	3.11 ± 0.23^##^	1.76 ± 0.10	0.59 ± 0.04	4.11 ± 0.50^#^	7.01 ± 0.48
HCBD/FSC-SpEt	3	5.96 ± 0.27	0.73 ± 0.03^##^	3.25 ± 0.10	1.51 ± 0.09	2.34 ± 0.18	0.47 ± 0.03	2.71 ± 0.24	8.33 ± 0.55
	9	5.91 ± 0.16	0.84 ± 0.05^#^	3.65 ± 0.18^##^	1.50 ± 0.09	2.49 ± 0.12^#^	0.41 ± 0.02^#^	2.26 ± 0.16^#^	8.43 ± 0.26
HCBD/FF	0.05	4.03 ± 0.16^##^	0.78 ± 0.04^##^	2.37 ± 0.08^##^	0.67 ± 0.06^##^	3.80 ± 0.39^##^	0.28 ± 0.02^##^	1.66 ± 0.15^##^	7.69 ± 0.30

Mice were fed with normal diet (ND) or high cholesterol/bile salt (1/0.3 %, w/w) diet (HCBD) to establish a model of mouse hypercholesterolemia (HCL). Fructus Schisandrae Chinensis seed (FSC-S) and post-ethanol extraction residue of FSC-S (FSC-SpEt) were smashed to pass through the 60 mesh sieve and mixed with either ND or HCBD at the indicated doses, which were estimated on the basis of crude herbal material. Fenofibrate (FF) was adopted as a positive control for comparison. Ten days after dietary supplementation, serum total cholesterol (TC), triglyceride (TG), high-density lipoprotein (HDL), low-density lipoprotein (LDL), non-HDL (N-HDL), and glucose (GLU) levels, as well as HDL/LDL and LDL/HDL ratio were measured. Values given are the means ± SEM, with n = 10. ^*^ *P* < 0.05, ^**^ *P* < 0.01 vs ND; ^#^ *P* < 0.05, ^##^ *P* < 0.01 vs HCBD. Statistical significant differences were determined using a one-way ANOVA followed by Dunnett’s multiple comparisons test or post-hoc analysis

HCBD-fed mice increased serum TC, LDL, and N-HDL levels (33, 317, and 102 %, respectively) as well as the LDL/HDL ratio (317 %), while serum TG level and HDL/LDL ratio were decreased by 23 and 76 %, respectively, when compared with those of normal mice. FSC-S supplementation significantly increased serum TC (64 %), TG (118 %), HDL (77 %) and LDL (107 %) levels in a dose-dependent manner in HCBD-fed mice. FSC-SpEt treatment increased both serum HDL (22 %) and HDL/LDL ratio (21 %), but decreased serum TG (15 %) and N-HDL (17 %) levels as well as LDL/HDL ratio (18 %) in HCL mice. FF treatment reduced serum TC (30 %), TG (21 %), HDL (21 %), LDL (55 %), and N-HDL (39 %) levels, but increased HDL/LDL ratio (85 %) in HCL mice (Table 1).

### Effects of FSC-S and FSC-SpEt supplementation on hepatic lipids and GLU

As shown in Table 2, the supplementation with FSC-S and FSC-SpEt decreased hepatic TC content by 19 % and 16 %, respectively, in normal mice. Supplementation with FSC-S increased hepatic TG (14 %) contents and decreased hepatic GLU (24 %) contents, whereas FSC-SpEt supplementation decreased hepatic TG contents (20 %). ND supplemented with FF reduced hepatic TC, TG, and GLU contents by 59, 50 and 28 %, respectively, when compared with the un-supplemented normal mice. Feeding mice with HCBD for 10 days markedly increased hepatic TC and TG contents by 57 and 63 %, respectively. When compared with un-supplemented HCL mice, the dietary consumption of FSC-S increased hepatic TG content (up to 20 %), and reduced hepatic GLU (up to 22 %) in a dose-dependent manner. Daily supplementation with FF lowered hepatic TC, TG and GLU contents by 64, 43, and 39 %, respectively (Table 2).

**Table 2:** Effects of diet supplementation with FSC-S and FSC-SpEt on hepatic lipids and GLU in normal and experimental hypercholesterolemic (HCL) mice

Groups	Dose	TC	TG	GLU
	(%, w/w)	(μmol/g)	(μmol/g)	(μmol/g)
*Normal mice*				
ND	–	3.94 ± 0.11	7.14 ± 0.15	132.98 ± 3.13
ND/FSC-S	9	3.21 ± 0.09^**^	8.14 ± 0.31^**^	100.86 ± 2.80^**^
ND/FSC-SpEt	9	3.31 ± 0.13^**^	5.73 ± 0.18^**^	138.95 ± 3.83
ND/FF	0.05	1.61 ± 0.06^**^	3.54 ± 0.25^**^	95.37 ± 3.67^**^
*HCL mice*				
ND	–	7.35 ± 0.08	6.82 ± 0.11	106.81 ± 3.83
HCBD	–	11.52 ± 0.55^**^	11.12 ± 0.82^**^	107.67 ± 2.88
HCBD/FSC-S	3	12.45 ± 0.39	13.05 ± 0.58	97.57 ± 2.35^#^
	9	11.90 ± 0.05	13.35 ± 0.48^#^	84.48 ± 1.65^##^
HCBD/FSC-SpEt	3	11.59 ± 0.51	10.13 ± 0.43	102.84 ± 1.67
	9	10.70 ± 0.47	9.70 ± 0.55	104.73 ± 1.67
HCBD/FF	0.05	7.02 ± 0.22^##^	6.34 ± 0.15^##^	65.67 ± 2.14^##^

Experimental details were described in Table 1. Mice were fed with ND and HCBD without or with FSC-S, FSC-SpEt, or FF supplementation. Ten days after supplementation, hepatic TC, TG and GLU contents were determined. Values given are the means ± SEM, with *n* = 10. ^*^ *P* < 0.05, ^**^ *P* < 0.01 vs ND; ^#^ *P* < 0.05, ^##^ *P* < 0.01 vs HCBD. Statistical significant differences were determined using a one-way ANOVA followed by Dunnett’s multiple comparisons test or post-hoc analysis

### Effects of FSC-S and FSC-SpEt supplementation on hepatic size and liver function

In normal mice, supplementations with FSC-S, FSC-SpEt, and FF caused an elevation in hepatic index by 56, 10, and 67 %, respectively, when compared with the un-supplemented control. Mice fed with HCBD for 10 days produced an increase in liver size by 16 % and serum alanine aminotransferase (ALT) activity by 22 %, when compared with the ND-fed mice. FSC-S, but not FSC-SpEt supplementation increased hepatic weight and index (up to 36 and 58 %, respectively) and serum ALT activity (approximately 33 %) in a dose-dependent manner. FF supplementation elevated hepatic weight, hepatic index, and serum ALT activity by 67, 71, and 78 %, respectively, in HCL mice (Table 3).

**Table 3:** Effects of FSC-S and FSC-SpEt supplementation on hepatic size and liver function in normal and experimental hypercholesterolemic (HCL) mice

Groups	Drug dose (%, w/w)	Hepatic weight (g)	Hepatic index	Serum ALT activity (U/L)
*Normal mice*				
ND	–	1.69 ± 0.06	5.98 ± 0.13	53.70 ± 4.30
ND/FSC-S	9	1.90 ± 0.13	9.32 ± 0.39^**^	42.83 ± 3.06
ND/FSC-SpEt	9	1.85 ± 0.04^*^	6.55 ± 0.08^**^	42.58 ± 3.75
ND/FF	0.05	2.75 ± 0.05^**^	10.00 ± 0.11^**^	46.59 ± 4.98
*HCL mice*				
ND	–	1.79 ± 0.06	6.11 ± 0.07	50.88 ± 1.30
HCBD	–	2.07 ± 0.05^**^	7.12 ± 0.09^**^	62.00 ± 3.59^*^
HCBD/FSC-S	3	2.56 ± 0.14^##^	8.81 ± 0.47^##^	73.78 ± 6.45
	9	2.87 ± 0.17^##^	11.28 ± 0.51^##^	82.63 ± 6.11^#^
HCBD/FSC-SpEt	3	1.99 ± 0.18	6.95 ± 0.15	71.50 ± 2.99
	9	2.01 ± 0.11	7.37 ± 0.13	63.25 ± 2.55
HCBD/FF	0.05	3.46 ± 0.08^##^	12.19 ± 0.20^##^	110.50 ± 13.06^##^

Experimental details were described in Table 1. Mice were fed with ND and HCBD without or with FSC-S, FSC-SpEt, or FF supplementation. Ten days after supplemetnation, hepatic weight and index, as well as serum alanine aminotransferase (ALT) activity were measured. Hepatic index was estimated from the ratio of its weight to body weight × 100. Values given are the means ± SEM, with *n* = 10. ^*^ *P* < 0.05, ^**^ *P* < 0.01 vs ND; ^#^ *P* < 0.05, ^##^ *P* < 0.01 vs HCBD. Statistical significant differences were determined using a one-way ANOVA followed by Dunnett’s multiple comparisons test or post-hoc analysis

### Effects of FSC-S and FSC-SpEt supplementation on organ/tissue weight/indices

Supplementation with both FSC-S and FF significantly decreased the epididymis fat weight and index in both normal and HCL mice. FSC-S supplementation also reduced kidney and spleen mass in both normal and HCL mice. For the normal mice, FSC-S did not change the testis weight but increased testis index 1 or index 2 by 32 and 39 %, respectively. However, FSC-SpEt supplementation did not alter these organ and epididymis fat weight and indices (Table 4).

**Table 4:** Effects of FSC-S and FSC-SpEt supplementation on organ/tissue mass in normal and experimental hypercholesterolemic (HCL) mice

Groups	Drug dose (%, w/w)	Weight (g)				Index 1				Index 2			
		Fat	Kindey	Spleen	Testis	Fat	Kindey	Spleen	Testis	Fat	Kindey	Spleen	Testis
*Normal mice*													
ND	–	0.25 ± 0.02	0.45 ± 0.02	0.14 ± 0.01	0.17 ± 0.01	0.90 ± 0.06	1.59 ± 0.06	0.51 ± 0.03	0.59 ± 0.04	0.96 ± 0.06	1.69 ± 0.06	0.54 ± 0.03	0.62 ± 0.04
ND/FSC-S	9	0.04 ± 0.01^**^	0.25 ± 0.01^**^	0.07 ± 0.01^**^	0.16 ± 0.01	0.19 ± 0.02^**^	1.22 ± 0.03^**^	0.36 ± 0.02^**^	0.78 ± 0.04^**^	0.21 ± 0.02^**^	1.35 ± 0.04^**^	0.39 ± 0.02^**^	0.86 ± 0.04^**^
ND/FSC-SpEt	9	0.24 ± 0.01	0.47 ± 0.01	0.13 ± 0.01	0.19 ± 0.01	0.83 ± 0.04	1.67 ± 0.03	0.45 ± 0.01	0.66 ± 0.02	0.89 ± 0.04	1.79 ± 0.03	0.49 ± 0.02	0.70 ± 0.02
ND/FF	0.05	0.12 ± 0.01^**^	0.44 ± 0.02	0.12 ± 0.01^*^	0.18 ± 0.01	0.45 ± 0.05^**^	1.59 ± 0.04	0.45 ± 0.02	0.66 ± 0.03	0.50 ± 0.05^**^	1.76 ± 0.05	0.50 ± 0.03	0.74 ± 0.03^*^
*HCL mice*													
ND	–	0.32 ± 0.02	0.48 ± 0.02	0.15 ± 0.01	0.20 ± 0.01	1.10 ± 0.05	1.65 ± 0.43	0.50 ± 0.02	0.69 ± 0.03	1.17 ± 0.05	1.76 ± 0.05	0.53 ± 0.02	0.74 ± 0.03
HCBD	–	0.23 ± 0.02^**^	0.48 ± 0.01	0.14 ± 0.01	0.20 ± 0.01	0.79 ± 0.06^**^	1.64 ± 0.04	0.50 ± 0.03	0.69 ± 0.03	0.85 ± 0.07^**^	1.77 ± 0.04	0.53 ± 0.03	0.74 ± 0.04
HCBD/FSC-S	3	0.26 ± 0.02	0.46 ± 0.02	0.15 ± 0.01	0.20 ± 0.01	0.88 ± 0.07	1.58 ± 0.05	0.51 ± 0.04	0.68 ± 0.02	0.97 ± 0.08	1.73 ± 0.05	0.56 ± 0.42	0.75 ± 0.02
	9	0.13 ± 0.02^##^	0.35 ± 0.01^##^	0.11 ± 0.01^##^	0.18 ± 0.01	0.49 ± 0.07^##^	1.39 ± 0.03^##^	0.44 ± 0.02	0.71 ± 0.04	0.56 ± 0.08^#^	1.57 ± 0.03^##^	0.49 ± 0.03	0.80 ± 0.05
HCBD/FSC-SpEt	3	0.21 ± 0.02	0.48 ± 0.02	0.14 ± 0.01	0.19 ± 0.01	0.71 ± 0.06	1.68 ± 0.06	0.50 ± 0.02	0.67 ± 0.03	0.77 ± 0.06	1.81 ± 0.07	0.53 ± 0.02	0.72 ± 0.04
	9	0.20 ± 0.02	0.48 ± 0.02	0.12 ± 0.01	0.20 ± 0.01	0.70 ± 0.06	1.70 ± 0.04	0.43 ± 0.02	0.70 ± 0.03	0.76 ± 0.07	1.84 ± 0.05	0.46 ± 0.03	0.75 ± 0.04
HCBD/FF	0.05	0.13 ± 0.01^##^	0.51 ± 0.20	0.12 ± 0.01^#^	0.20 ± 0.01	0.45 ± 0.02^##^	1.79 ± 0.07	0.41 ± 0.03^#^	0.71 ± 0.02	0.51 ± 0.02^##^	2.03 ± 0.08^##^	0.47 ± 0.03	0.80 ± 0.02

Experimental details were described in Table 1. Mice were fed with ND and HCBD without or with FSC-S, FSC-SpEt, or FF supplementation. Ten days after supplementation, epididymis fat, kidney, spleen and testis mass and index were measured. Index 1 and index 2 were estimated from the ratio of their weight to body weight × 100 or their weight to body weight – liver weight × 100, respectively. Values given are the means ± SEM, with n = 10. ^*^ *P* < 0.05, ^**^ *P* < 0.01 vs ND; ^#^ *P* < 0.05, ^##^ *P* < 0.01 vs HCBD. Statistical significant differences were determined using a one-way ANOVA followed by Dunnett’s multiple comparisons test or post-hoc analysis

### Effects of FSC-S and FSC-SpEt supplementation on stomach weight/indices, residual gastric contents and fecal TC contents

Feeding mice with ND-supplemented FSC-S significantly reduced stomach weight by 26 %, but it increased residual gastric content and both index 1 and 2 by 33, 87, or 94 %, respectively. Supplementation with FSC-SpEt showed no effect on stomach weight and residual gastric content or indices, but it decreased fecal TC level by 18 %. FF supplementation reduced stomach weight and index 1 by 18 and 14 %, respectively. In HCL mice, fecal TC level was significantly increased by 342 %. FF supplementation increased fecal TC level by 20 % and stomach index 2 by 12 % in HCBD mice, when compared with un-supplemented HCBD mice. FSC-S treatment increased stomach index 2 (13 %) and residual gastric content index 1 (41 %) and 2 (48 %) in HCL mice. FSC-SpEt supplementation decreased residual gastric content and the index (up to 32 %) in HCL mice (Table 5).

**Table 5:** Effects of FSC-S and FSC-SpEt supplementation on stomach and residual gastric content and fecal TC excretion in normal and experimental hypercholesterolemic (HCL) mice

Groups	Drug dose (%, w/w)	Stomach			Residual gastric content			Fecal TC (μmol/g)
		Weight (g)	Index 1	Index 2	Weight (g)	Index 1	Index 2	
*Normal mice*								
ND	–	0.34 ± 0.02	1.18 ± 0.06	1.26 ± 0.06	0.55 ± 0.06	1.92 ± 0.20	2.05 ± 0.21	5.91 ± 0.22
ND/FSC-S	9	0.25 ± 0.02^**^	1.25 ± 0.06	1.38 ± 0.07	0.73 ± 0.06^*^	3.59 ± 0.20^**^	3.97 ± 0.23^**^	5.21 ± 0.57
ND/FSC-SpEt	9	0.32 ± 0.01	1.12 ± 0.03	1.20 ± 0.03	0.45 ± 0.05	1.59 ± 0.19	1.70 ± 0.20	4.82 ± 0.29^**^
ND/FF	0.05	0.28 ± 0.02^*^	1.02 ± 0.05^*^	1.31 ± 0.06	0.42 ± 0.03	1.53 ± 0.10	1.70 ± 0.11	6.72 ± 0.40
*HCL mice*								
ND	–	0.31 ± 0.01	1.06 ± 0.03	1.13 ± 0.03	0.61 ± 0.09	2.06 ± 0.26	2.20 ± 0.28	5.24 ± 0.47
HCBD	–	0.30 ± 0.01	1.05 ± 0.03	1.12 ± 0.03	0.53 ± 0.06	1.82 ± 0.20	1.96 ± 0.22	23.18 ± 1.00^**^
HCBD/FSC-S	3	0.29 ± 0.01	0.99 ± 0.04	1.08 ± 0.04	0.49 ± 0.09	1.70 ± 0.32	1.88 ± 0.37	23.72 ± 0.80
	9	0.28 ± 0.01	1.12 ± 0.04	1.26 ± 0.04^#^	0.66 ± 0.08	2.57 ± 0.27^#^	2.91 ± 0.31^#^	20.86 ± 1.06
HCBD/FSC-SpEt	3	0.31 ± 0.02	1.08 ± 0.06	1.16 ± 0.06	0.36 ± 0.02^#^	1.27 ± 0.07^#^	1.37 ± 0.08^#^	25.43 ± 1.72
	9	0.30 ± 0.01	1.07 ± 0.04	1.15 ± 0.04	0.41 ± 0.04^#^	1.41 ± 0.12	1.53 ± 0.13	24.87 ± 0.96
HCBD/FF	0.05	0.31 ± 0.01	1.10 ± 0.04	1.25 ± 0.04^#^	0.37 ± 0.04^#^	1.29 ± 0.11^#^	1.47 ± 0.12	27.84 ± 1.19^##^

Experimental details were described in Table 1 and 3. Mice were fed with ND and HCBD without or with FSC-S, FSC-SpEt, or FF supplementation. Ten days after supplementation, stomach weight and the values of gastric content and fecal TC were measured. Stomach and residual gastric content index was estimated from the ratio of their weight to body weight × 100 (index 1) or the ratio of their weight to body weight – liver weight × 100 (index 2). Values given are the means ± SEM, with *n* = 10. ^*^ *P* < 0.05, ^**^ *P* < 0.01 vs ND; ^#^ *P* < 0.05, ^##^ *P* < 0.01 vs HCBD. Statistical significant differences were determined using a one-way ANOVA followed by Dunnett’s multiple comparisons test or post-hoc analysis

### Effects of FSC-S and FSC-SpEt supplementation on body weight

FSC-S supplement suppressed the increase in body weight (approximately 28 % in normal mice and 13 % in HCL mice), when compared with the corresponding normal and HCL mice without FSC-S supplementation. FSC-S and FF reduced the values of body weight minus liver weight by 31 and 7 % in normal mice and 17 and 7 % in HCL mice, respectively. FSC-SpEt did not alter the body weight gain in both mice (Table 6).

**Table 6:** Effects of FSC-S and FSC-S_pEt_ supplement on body weight (BW) gain in normal and experimental hypercholesterolemic (HCL) mice

Groups	Drug dose (%, w/w)	BW (g)	BW gain (g)			BW (g) at	BW-LW (g) at D10
		at D0	D2-D0	D6-D0	D10-D0	D10	
*Normal mice*							
ND	–	17.10 ± 0.23	3.99 ± 0.76	8.13 ± 0.49	11.17 ± 0.54	28.27 ± 0.47	26.57 ± 0.43
ND/FSC-S	9	17.41 ± 0.22	-0.92 ± 0.41^**^	0.71 ± 0.56^**^	2.80 ± 0.86^**^	20.22 ± 0.73^**^	18.32 ± 0.62^**^
ND/FSC-S_pEt_	9	17.21 ± 0.21	3.87 ± 0.30	8.33 ± 0.41	11.05 ± 0.34	28.26 ± 0.26	26.41 ± 0.23
ND/FF	0.05	17.33 ± 0.23	3.82 ± 0.46	7.33 ± 0.59	10.21 ± 0.69	27.54 ± 0.55	24.79 ± 0.51^*^
*HCL mice*							
ND	–	20.74 ± 0.40	2.73 ± 0.52	5.90 ± 0.70	8.45 ± 0.71	29.19 ± 0.68	27.40 ± 062
HCBD	–	20.73 ± 0.28	2.41 ± 0.45	5.88 ± 0.39	8.25 ± 0.49	28.99 ± 0.47	26.92 ± 0.48
HCBD/FSC-S	3	20.52 ± 0..31	2.87 ± 0.55	6.45 ± 0.70	8.57 ± 0.64	29.09 ± 0.68	26.53 ± 0.63
	9	20.90 ± 0.37	-0.78 ± 0.41^##^	2.06 ± 0.60^##^	4.41 ± 0.79^##^	25.31 ± 0.61^##^	22.44 ± 0.49^##^
HCBD/FSC-S_pEt_	3	20.29 ± 0.35	2.87 ± 0.38	6.31 ± 0.48	8.37 ± 0.69	28.66 ± 0.54	26.66 ± 0.50
	9	20.71 ± 0.34	2.61 ± 0.40	5.53 ± 0.85	7.71 ± 0.72	28.42 ± 0.79	26.32 ± 0.70
HCBD/FF	0.05	20.67 ± 0.35	3.29 ± 0.39	6.31 ± 0.41	7.74 ± 0.54	28.40 ± 0.46	24.94 ± 0.40^##^

Experimental details were described in Table 1. Mice were fed with ND and HCBD without or with FSC-S, FSC-S_pEt_, or FF supplementation. Body weight (BW) in each mouse was measured before medication (D0) and at D2, D6 and D10 following administration of drug. In addition, the values of BW minus liver weight (LW) were also determined due to hepatomegaly caused by FSC-S and FF supplement. Values given are the means ± SEM, with *n* = 10. ^*^ *P* < 0.05, ^**^ *P* < 0.01 vs ND; ^#^ *P* < 0.05, ^##^ *P* < 0.01 vs HCBD. Statistical significant differences were determined using a one-way ANOVA followed by Dunnett’s multiple comparisons test or post-hoc analysis

### Effects of FSC-S and FSC-SpEt supplementation on food, water, and drug intake

As shown in Table 7, the ratio of food and water intake to body weight (RFBW and RWBW, respectively) and feed efficiency index (FEI) and water efficiency index (WEI) values showed no detectable differences between normal and HCL mice. While FSC-S supplementation at the dose of 9 % reduced RFBW by 18 % in both normal and HCL mice, it elevated FEI and WEI by 133 and 95 % in normal mice and 33 and 41 % in HCL mice, respectively. However, FSC-SpEt and FF supplementations did not alter the daily food/water intake in normal and HCL mice. Daily intake of FSC-S/FSC-SpEt was estimated to be 5.13/5.44 g/kg (based on crude herb equivalent) and 12.73-12.79/16.76-17.93 g/kg at 3 % and 9 % supplementation, respectively. FF was estimated to be 0.08 and 0.1 g/kg/day in ND-fed and HCL mice, respectively.

**Table 7:** Effects of FSC-S and FSC-SpEt supplementation on food, water and drug intake in normal and experimental hypercholesterolemic (HCL) mice

Groups	Drug dose (%, w/w)	Food intake (g/kg/day)	RFBW	FEI	Water intake (ml/kg/day)	RWBW	WEI	Drug intake (g/kg/day)
*Normal mice*								
ND	–	176.74	1.56	3.95	310.90	2.94	7.44	–
ND/FSC-S	9	130.09	1.28	9.21	222.57	2.01	14.50	12.73
ND/FSC-SpEt	9	169.48	1.66	4.24	276.40	1.06	6.29	16.76
ND/FF	0.05	167.29	1.49	4.03	287.38	2.56	6.92	0.08
*HCL mice*								
ND	–	184.89	1.67	5.78	269.88	2.44	8.44	–
HCBD	–	174.75	1.57	5.53	271.64	2.47	8.68	–
HCBD/FSC-S	3	165.93	1.57	5.33	257.95	2.37	8.04	5.13
	9	129.31	1.28	7.36	236.87	2.14	12.27	12.79
HCBD/FSC-SpEt	3	176.02	1.67	5.72	271.91	2.50	8.57	5.44
	9	181.26	1.83	6.75	244.30	2.24	8.28	17.93
HCBD/FF	0.05	192.56	1.81	6.63	265.16	2.49	9.13	0.10

Experimental details were described in Table 1. Mice were fed with ND and HCBD without or with FSC-S, FSC-SpEt, or FF supplementation for 10 days. During supplementation, the volumes of food/water/drug intake and the ratio of food/water intake to body weight were estimated in each group. The dosages of drug intake (g/kg/day) were determined by noting the amount of ingested diet (g/kg/day) and the drug concentrations in the diet. The weight of drug was subtracted from the food intake. FSC-S and FSC-SpEt doses were based on their crude herbal material. The ratio of food (g) and water (mL) intake to body weight (g) (RFBW and RWBW, respectively) was calculated by using the formula as follow: total food (g) and water (mL) intake divided total body weight (g) for an experimental period of 10 days. The feed efficiency index (FEI) and water efficiency index (WEI) was calculated by using the formula as follow: food (g) and water (mL) consumption divided body weight gain (g) for the 10 days of treatment with drugs

## Discussion

It is well known that dietary intake is causally related to hyperlipidemia, including hypercholesterolemia, hypertriglyceridemia and their combination in experimental animals and humans [25–27]. In the present study, feeding mice with HCBD for 10 days caused significant increases in serum TC and LDL levels, but serum TG level was significantly decreased. This is consistent with observations in our previous study [20, 21]. However, it has been shown that elevations in serum TC and TG occur in rabbits fed a diet supplemented with 1 % cholesterol for 60 days [28]. The degree of cholesterol-induced hypertriglyceridemia may vary among animal species, which may be related to the duration of high cholesterol intake. Currently, serum parameters of N-HDL, LDL/HDL and HDL/LDL have been clinically used for predicting the risk of CVD in patients with dyslipidemia, as well as monitoring the effectiveness of lipid-lowering agent in patients [29–31]. As observed in the present study, HCBD-fed mice showed a decrease in serum HDL/LDL and increases in serum N-HDL and LDL/HDL values. This animal model may be appropriately used for evaluating the effectiveness of drug on serum lipid profile, which is predictive of risk on CVD. FSC-S supplementation was found to increase serum lipid levels, including those of “good cholesterol” and “bad cholesterol”, in both normal and HCL mice. While FSC-SpEt increased serum HDL level and HDL/LDL ratio, it decreased serum TG, LDL, and N-HDL levels. Currently, it is widely believed that bad cholesterol plays an important role in the development of CVD. In contrast, good cholesterol is responsible for returning excessive cholesterol in the bloodstream back to the liver where it is excreted in bile. Given the beneficial effect of FSC-SpEt on serum lipid profile, it may be potentially used for alternative treatment in patients with hyperlipidemia.

The liver plays a key role in lipid metabolism. Feeding mice with HCBD for 10 days increased hepatic lipid accumulation and size, which was associated with liver injury, as evidenced by an increase in serum ALT activity. Although both FSC-S and FSC-SpEt supplementations did not reduce hepatic lipid content in HCL mice, they lowered hepatic TC contents in normal mice. FSC-S supplementation also decreased hepatic GLU content in both normal and HCL mice. The ability of FSC-S and FSC-SpEt supplementation to elevate serum TC levels and lower hepatic TC content may be related to the release of hepatic TC into bloodstream in supplemented mice. Given that lignans presented in FSC could improve insulin sensitivity via activating the peroxisome proliferator-activated receptor (PPAR)-γ pathways [15]. The supplementation with FSC-S, but not FSC-SpEt (which is deprived of lignans), reduced serum and hepatic GLU levels in mice. It has been demonstrated that FSC has hepatoprotective activity [13]. However, serum ALT activity was slightly elevated in the FSC-S-supplemented HCL mice. This observation may be related to the increase in liver size in HCL mice.

While FSC-S supplementation decreased body weight, epididymis fat, kidney and spleen weight and indices in normal and HCL mice, FSC-SpEt only induced an increase in liver size in normal mice. The observation that losses of body weight and fat occurred in FSC-S supplemented mice suggests the utilization of FSC-S as a prophylactic or therapeutic agent in overweight or obese individuals. Although the amount of food intake and the ratio of food intake to metabolic body mass as indicated by RFBW were reduced in both FSC-S/normal and FSC-S/HCL mice, their FEI values markedly increased as compared with the corresponding un-supplemented animals. This indicated that some ingested food/energy is not converted into body mass in both normal and HCL mice supplemented with FSC-S. FEI (weight gain per unit of food ingested) is an index reflecting the amount of feed to produce one kilogram of meat in animal husbandry industry [32–36]. In this study, FEI reflects the FSC-S-induced body weight loss in normal and HCL mice.

FF, a synthetic fibric acid derivative, is a prescribed drug for the treatment of dyslipidemia. The therapeutic effect of FF, which is mediated by PPAR-α activation, is consisted of reduction in the blood TC and LDL, especially TG levels, as well as the associated increase in HDL [37, 38]. In the present study, FF supplementation decreased serum TC, TG, LDL and N-HDL levels, but it increased HDL/LDL in HCL mice. In addition, FF also lowered hepatic TC, TG and GLU levels in both normal and HCL mice. Despite the fact that FF produces a significant effect on the metabolism of lipids as well as GLU, including down-regulation of lipogenesis and up-regulation of fatty acid oxidation [39], the liver injury, as indicated by high serum ALT activity and hepatomegaly, was aggravated by FF treatment in HCL mice. This is consistent with the earlier experimental and clinical observations [20, 21, 40–42].

Chinese herbal medicine (CHM) has been used for over 2000 years in China. Since the introduction of Western medicine in China during the 16^th^ century, CHM has become an alternative medicine rather than the mainstream medicine in China [43]. In recent decades, however, more and more people suffer from chronic diseases which cannot be treated effectively with chemical drugs in Western medicine. Consequently, complementary and alternative medicine, including CHM, has gained more attention and thus becomes popular [44, 9]. Today, about 80 % of people worldwide rely on herbal medicines for some aspects of their primary health care, and more than 8000 varieties of CHM or related herbal products are now exported from China to more than 130 countries and regions [44, 11]. The increasing demand for herbal products in the global market would likely pose tremendous challenge on herbal resources in the world. For example, the Panax herb (eg. ginseng) was being consumed by millions and millions of people around the world, while wild *Panax japonicus* plants have become increasingly rare in China [45]. The effects of crude FSC-S (without any processing) and FSC-SpEt (usually treated as waste in the modern pharmaceutical industry) were compared in normal and HCL mice in the present study which aimed to demonstrate how natural herbal material can be fully utilized. The research and development of herbal medicine, while under the guidance of scientific principle, should be economical, simple, safe, effective, and environmental friendly. Results obtained from the present study showed that both crude herb FSC-S and its “waste” FSC-SpEt possess a variety of bioactivities resembling its “active extract” or “active compound”.

In conclusion, results obtained from the present study suggested that daily supplementation with FSC-S and FSC-SpEt may provide an effective alternative intervention for the management of hyperlipidemia/fatty liver and overweight/obesity, respectively. Data are summarized in Table 8.

**Table 8:** A summary on the effects of FSC-S and FSC-SpEt supplementation in normal and experimental hypercholesterolemic (HCL) mice

		FSC-S FSC-SpEt		FF			FSC-S		FSC-SpEt		FF
		9 %	9 %				3 %	9 %	3 %	9 %	
*Vs normal mice*					*Vs HCL mice*						
Serum	TC	↑	↑	—	Serum	TC	↑	↑	—	—	↓
	TG	↑	—	↓		TG	—	↑	↓	↓	↓
	HDL	↑	—	—		HDL	↑	↑	—	↑	↓
	LDL	↑	↑	—		LDL	↑	↑	—	—	↓
	ALT activity	—	—	—		ALT activity	—	↑	—	—	↑
	LDL/HDL	—	↑	—		LDL/HDL	↑	—	—	↓	↓
	HDL/LDL	—	↓	—		HDL/LDL	↓	—	—	↑	↑
	N-HDL	↑	↑	—		N-HDL	—	↑	—	↓	↓
Hepatic	TC	↓	↓	↓	Hepatic	TC	—	—	—	—	↓
	TG	↑	↓	↓		TG	—	↑	—	—	↓
	Glucose	↓	—	↓		Glucose	↓	↓	—	—	↓
Body	weight	↓	—	—	Body	weight	—	↓	—	—	—
BW -	liver weight	↓	—	↓	BW -	liver weight	—	↓	—	—	↓
Liver	index/weight	—/↑	↑/↑	↑/↑	Liver	index/weight	↑/↑	↑/↑	—/—	—/—	↑/↑
Fat	index/weight	↓/↓	—/—	↓/↓	Fat	index/weight	—/—	↓/↓	—/—	—/—	↓/↓
Kidney	index/weight	↓/↓	—/—	—/—	Kidney	index/weight	—/—	↓/↓	—/—	—/—	↑/—
Spleen	index/weight	↓/↓	—/—	—/↓	Spleen	index/weight	—/—	—/↓	—/—	—/—	↓/↓
Testis	index/weight	↑/—	—/—	—/—	Testis	index/weight	—/—	—/—	—/—	—/—	—/—
Stomach	index/weight	—/↓	—/—	↓/↓	Stomach	index/weight	—/—	↑/—	—/—	—/—	↑/—
Gas-content	index/weight	↑/↑	—/—	—/—	Gas-content	index/weight	—/—	↑/—	↓/↓	—/↓	↓/↓
Fecal	TC	—	↓	—	Fecal	TC	—	—	—	—	↑

↑: increased or elevated; ↓: decreased or inhibited; —: unaltered

## Materials and methods

### Preparation of FSC-S and FSC-SpEt

FSC was purchased from Guangzhou ZhiXin herbal Co., Ltd and authenticated by Professor Chun-Sheng Liu at Beijing University of Chinese Medicine. FSC were manually peeled off and its seeds were washed with distilled water for removing the residual pulps. For the preparation of post-ethanol extraction FSC seeds, the dried seeds were crushed into small pieces using an industrial grinder and extracted twice (first, 1.5 h; second, 2 h) with 5 volumes of 80 % (v/v, in H_2_O) ethanol under reflux after soaking for half an hour. FSC-SpEt was dried at room temperature. Both FSC-S and FSC-SpEt were smashed to pass through the 60 mesh sieve and stored at 4 °C until use.

### Chemicals and regents

FF (certificate number 20667) was bought from Beijing Jinxiang Medical Ltd (Beijing, People’s Republic of China). Cholesterol (certificate number 20120614) and bile salt (certificate number 20121210) were purchased from Beijing Chemical Reagent Co (Beijing, People’s Republic of China). Assay kits for TC (certificate number 141721), TG (certificate number 146781), and GLU (certificate number 143291) were supplied by Zhongsheng Beikong Biotechnology and Science Inc. (Beijing, People’s Republic of China). Assay kits for HDL, LDL and ALT were bought from Zhongsheng Beikong Bio-technology and Science Inc. (Beijing, People’s Republic of China) or Beijing Leadman Biochemistry Co Ltd (Beijing, People’s Republic of China).

### Animal and treatment

Male ICR (grade II, certificate number SCXK [jing] 2012-0001) mice weighing 17—21 g were obtained from Vital River Lab Animal Co Ltd (Beijing, People’s Republic of China). Animals were housed under controlled conditions (20 °C—22 °C, relative humidity 50—55 %, 12:12-h dark-light cycle) with free access to regular chow and water. Blood and liver samples were obtained from ether-anesthetized animals that had been fasted for 6 h (from 6 am to 12 noon) and were subjected to biochemical analysis. All experimental procedures were approved by the University Committee on Research Practice at the Beijing University of Chinese Medicine.

### Experimental design

#### Design 1

In this study, the effects of FSC-S and FSC-SpEt supplementation on lipids, GLU, and liver function were investigated in normal mice. Animals were divided into four groups of 10 animals in each: Group 1, mice fed with ND; Group 2, 3, 4, mice fed with ND supplemented with 9 % FSC-S, 9 % FSC-SpEt, and 0.05 % FF (w/w), respectively. After 10 days of supplementation, mice were sacrificed under light ether anesthesia. Blood samples were collected from the orbital vein, and liver tissue samples were obtained and subjected to biochemical analysis.

#### Design 2

**Fig. 1:**
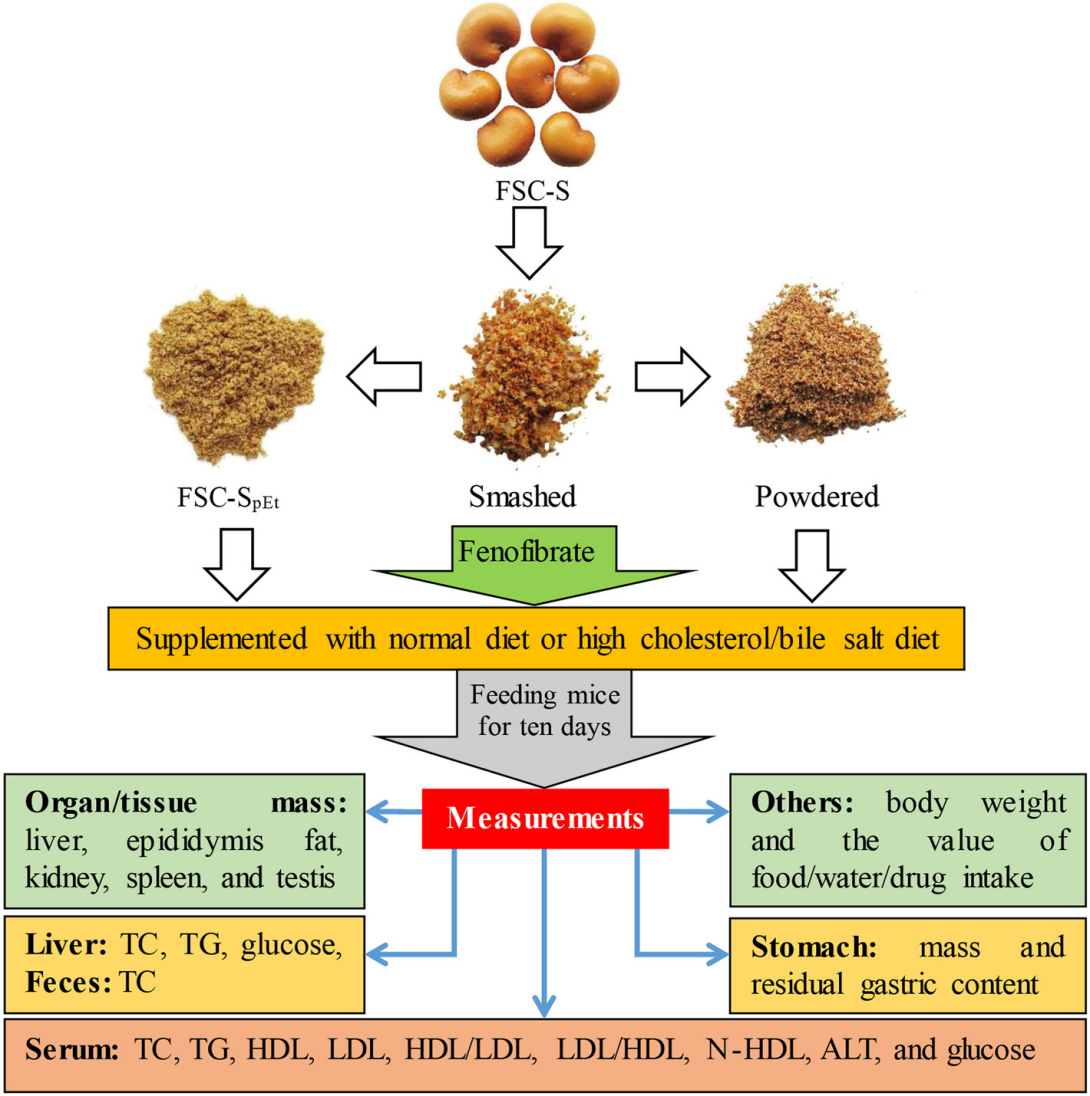
Experimental design of studies on FSC-S and FSC-SpEt in normal and HCL mice. *Abbreviations:* FSC-S: Fructus Schisandrae Chinensis seed; FSC-SpEt: post-ethanol extraction residue of FSC-S; TC: total cholesterol; TG: triglyceride; LDL: low-density lipoprotein; HDL: high-density lipoprotein; N-HDL: non-HDL; ALT: alanine aminotransferase; HCL: hypercholesterolemia

This study was designed to investigate the effects of FSC-S and FSC-SpEt supplementation on serum and hepatic parameters in HCL mice. Animals were randomly divided into four groups of 10 animals in each: Group 1, mice fed with ND; Group 2, mice fed with high cholesterol/bile salt (1/0.3 %, w/w) diet (HCBD); Group 3, 4, 5, and 6, mice fed with HCBD supplemented with FSC-S (3 %, 9 %) and FSC-SpEt (3 %, 9 %), respectively; Group 7, mice fed with HCBD supplemented with 0.05 % FF. After 10 days of supplementation, mice were sacrificed under light ether anesthesia. Blood samples were collected from the orbital vein, and liver tissue samples were obtained and subjected to biochemical analysis. Figure 1 shows the design of this study.

### Preparation of serum and hepatic supernatant fractions

Blood samples were collected from the orbital vein and centrifuged at 3000 × *g* for 8 min, and stored at −80 °C until used for biochemical analysis. Liver tissue samples were homogenized in 9 volumes of 0.9 % (w/v) NaCl solution by two 10 s bursts of a tissue disintegrator at 13,500 rpm and then stored at −20 °C until analysis within 48 h. The liver homogenates were centrifuged at 3500 × *g* for 15 min to obtain the supernatant fraction for lipid determination.

### Biochemical analysis

Ten μL serum and 30 μL hepatic supernatant were used to assay for TG and TC levels with glycerol phosphate oxidase-p-aminophenazone (GPO-PAP) and cholesterol oxidase phenol 4-aminoantipyrine peroxidase (COD-PAP) methods, respectively. Ten μL serum and hepatic supernatant were used to determine the GLU levels with GLU oxidase-peroxidase (GOD-POD) method. Mouse feces were collected on D10 and dried at room temperature. The dried feces (approximately 30 mg) were smashed and extracted with 0.5 mL chloroform-methanol (1:1, v/v) mixture for 12 h and then centrifuged at 2000 × *g* for 5 min to obtain the supernatants. Aliquots (30 μL) of fecal supernatants were used to measure the TC levels by using the COD-PAP method. All methods were performed according to the manufacturer's instructions. An automatic biochemistry analyzer (Synchron CX4 PRO; Beckman Coulter, Brea, CA, USA) was used to automatically measure the serum HDL and LDL levels and the ALT activity by a colorimetric method. In addition, serum non-HDL (N-HDL) levels were calculated as: TC – HDL.

### Measurement of body weight and organ indices

Body weight was measured every two days. At D10, the liver, epididymal fat, spleen, kidney, testis, and stomach, as well as residual gastric content were weighed. Organ/residual gastric content index 1 was calculated as organ weight or residual gastric content/body weight × 100. Organ/residual gastric content index 2 was calculated as [organ or residual gastric content/(body weight – liver weight)] × 100.

### Measurement of RFBW, RWBW, FEI, and WEI

RFBW and RWBW were calculated by using the formula as follow: total food (g) or water (mL) intake was divided by the total body weight (g) within the 10-day supplementation period. FEI and WEI were calculated by using the formula as follow: food (g) or water (mL) consumption divided by the body weight gain (g) for the duration of 10 days of supplementation with FSC-S, FSC-SpEt, or FF.

### Statistical analysis

The results are presented as the means ± SEM. Data were analyzed by one-way ANOVA. Comparisons between two groups were performed using the Dunnett’s multiple comparisons test or post-hoc analysis. Differences were considered significant at *P* < 0.05. Statistical tests of data were performed using the SPSS 20.0 statistical analysis program.

## Abbreviations

FSC-S: Fructus Schisandrae Chinensis seed
FSC-SpEt: post-ethanol extraction residue of FSC-S
FF: fenofibrate
CHM: Chinese herbal medicine
TC: total cholesterol
TG: triglyceride
LDL: low-density lipoprotein
HDL: high-density lipoprotein
N-HDL: non-HDL
ALT: alanine aminotransferase
HCL: hypercholesterolemia
CVD: cardiovascular diseases
RFBW: ratio of food intake to body weight
RWBW: ratio of water intake to body weight
FEI: feed efficiency index
WEI: water efficiency index
ND: normal diet
HCBD: high cholesterol/bile salt diet
